# StemnesScoRe: an R package to estimate the stemness of glioma cancer cells at single-cell resolution

**DOI:** 10.55730/1300-0152.2672

**Published:** 2023-12-15

**Authors:** Necla KOÇHAN, Yavuz OKTAY, Gökhan KARAKÜLAH

**Affiliations:** 1 İzmir Biomedicine and Genome Center, İzmir, Turkiye; 2 İzmir International Biomedicine and Genome Institute, Dokuz Eylül University, İzmir, Turkiye; 3 Department of Medical Biology, Faculty of Medicine, Dokuz Eylül University, İzmir, Turkiye

**Keywords:** Glioblastoma, stemness score, scATAC-seq, machine learning

## Abstract

**Background/aim:**

Glioblastoma is the most heterogeneous and the most difficult-to-treat type of brain tumor and one of the deadliest among all cancers. The high plasticity of glioma cancer stem cells and the resistance they develop against multiple modalities of therapy, along with their high heterogeneity, are the main challenges faced during treatment of glioblastoma. Therefore, a better understanding of the stemness characteristics of glioblastoma cells is needed. With the development of various single-cell technologies and increasing applications of machine learning, indices based on transcriptomic and/or epigenomic data have been developed to quantitatively measure cellular states and stemness. In this study, we aimed to develop a glioma-specific stemness score model using scATAC-seq data for the first time.

**Materials and methods:**

We first applied three powerful machine-learning algorithms, i.e. random forest, gradient boosting, and extreme gradient boosting, to glioblastoma scRNA-seq data to discover the most important genes associated with cellular states. We then identified promoter and enhancer regions associated with these genes. After downloading the scATAC-seq peaks and their read counts for each patient, we identified the overlapping regions between the single-cell peaks and the peaks of genes obtained through machine-learning algorithms. Then we calculated read counts that were mapped to these overlapping regions. We finally developed a model capable of estimating the stemness score for each glioma cell using overlapping regions and the importance of genes predictive of glioblastoma cellular states. We also created an R package, accessible to all researchers regardless of their coding proficiency.

**Results:**

Our results showed that mesenchymal-like stem cells display higher stemness scores compared to neural-progenitor-, oligodendrocyte-progenitor-, and astrocyte-like cells.

**Conclusion:**

scATAC-seq can be used to assess heterogeneity in glioblastoma and identify cells with high stemness characteristics. The package is publicly available at https://github.com/Necla/StemnesScoRe and includes documentation with implementation of a real-data experiment.

## 1. Introduction

Glioblastoma (GBM), also known as glioblastoma multiforme, is the most common aggressive brain tumor among primary brain tumors. Its treatment poses significant challenges and complexities. Although the exact cause of glioblastoma tumors has not been clearly established, researchers have identified certain risk factors, including ionizing radiation, some viral infections such as the Epstein–Barr virus and cytomegalovirus, advanced age, male sex, some ethnicities, and genetic background. Interestingly, a history of allergy (respiratory allergies, eczema, and asthma) has been shown to be a protective factor. While GBM accounts for 1.4% of all cancer types, it accounts for 2.9% of cancer-related deaths (Özduman et al., 2019). GBM patients who undergo surgical treatment have an average lifespan range of 12 to 18 months, which can vary with age, sex, and country of residence (Delgado López and Corrales-García, 2016).

Although there are many studies on brain cancer research, a treatment that completely cures GBM tumors has not been found yet. The treatment of GBM patients involves surgical intervention, radiotherapy, and chemotherapy. One of the biggest challenges in treatment is the resistance of glioblastoma tumor cells, which have a heterogeneous structure, to drugs and/or therapy. It is suggested that this resistance to treatment arises from glioma stem cells, which are one of the heterogeneous cell groups that form GBMs (Alagoz, 2018; Eberhart and Bar, 2020; Suvà and Tirosh, 2020).

With the development of next generation sequencing technologies, the complex and heterogeneous structure of many cancer types has been more extensively studied, resulting in a greater understanding of cancer cells. The use of single-cell sequencing technologies, which have become increasingly popular in recent years, has enabled the investigation of tumor cell heterogeneity at the single-cell resolution (Tang et al., 2009). These technologies play an important role in identifying different cell populations, measuring the frequency of cell types in tissues, characterizing differences in similar cell types, and investigating population heterogeneity (Andrews and Hemberg, 2018). Furthermore, recent advances in molecular and computational biology have extended single-cell sequencing beyond classical transcriptomic profiling at the cellular level. As a result, different data on the genomic and epigenomic properties of a single cell have been obtained. For example, the transposase-accessible chromatin with high throughput sequencing, which determines accessible regions in chromatin and helps us understand the epigenetic heterogeneity of complex tissue structures at single-cell resolution, has become a frequently used method in recent years and has gained momentum in the last few years.

As a result of the development of next-generation sequencing technologies, the heterogeneous structure of tumors has begun to be understood and it has been revealed that there are different cell populations in the same tumor tissue. Cancer stem cells are one of these populations and the study of cancer stem cells, which are thought to play an active role in the growth and recurrence of tumor cells, is critical in understanding their initiation, development, and resistance to cancer treatment. Therefore, new stemness indices have been defined, such as the DNA methylation-based stemness index (mDNAsi) and mRNA expression-based stemness index (mRNAsi), to be used in determining stemness-related features associated with tumor recurrence (Malta et al., 2018; Zhang et al., 2020). These indices, calculated independently of each other, were obtained using a machine learning algorithm called the one-class logistic regression model. High values obtained for stemness indices have been associated with biological processes that are active in cancer stem cells and tumor dedifferentiation (Malta et al., 2018). In addition, the literature shows that there are entropy-based approaches that express stemness index as transcriptomic stemness (Grün et al., 2016; Guo et al., 2017; Teschendorff and Enver, 2017). However, all of these approaches primarily use transcriptomic data, and do not adequately leverage epigenomic data at all or use it insufficiently. An approach that uses scATAC-seq data, which measures “chromatin accessibility”, one of the most important determinants of gene expression control and one that has been increasingly used in single-cell analyses in recent years, to determine a stemness index has not yet been developed.

While next-generation sequencing technologies are rapidly developing, performing both scRNA-seq (single-cell RNA sequencing) and scATAC-seq (single-cell ATAC sequencing) analyses for the same GBM samples is not currently a practical approach in terms of cost. Therefore, unlike the approaches developed to determine the stemness index, which may not be practical, in this project we aimed to develop a new stemness score specific to glioblastoma using scATAC-seq data. In this way, researchers will be able to investigate the cancer stemness of GBM cells using only accessible regions in chromatin (scATAC-seq data). An R package called StemnesScoRe was also developed and the source code implementing the method is available at https://github.com/Necla/StemnesScoRe/.

## 2. Materials and methods

### 2.1. scRNA-seq data

To find marker genes that can differentiate cellular types of GBM, we used scRNA-seq data downloaded from https://www.ncbi.nlm.nih.gov/bioproject/ under the accession number PRJNA324289. These data are composed of 329 high quality single cells originating from four adult cell populations: astrocytes (34 cells), quiescent NSCs (879 cells) and activated NSCs (152 cells), and more committed NPCs (64 cells). These cells were freshly isolated from young adult mouse subventricular zones. For a comprehensive understanding of these data and insights into the sequencing procedure employed, readers are encouraged to refer to Dulken et al. (2017).

### 2.2. Application of machine learning methods

In this part of the study, we applied three powerful machine learning algorithms: random forest (RF), gradient boosting (GB), and extreme gradient boosting (XGB). These are robust and efficient classification algorithms for high-dimensional data. To apply these ML algorithms, the data are divided into two parts, with 70% of the data used as training data and 30% used as test data. This ratio was preserved for each subtype. Each classification algorithm was applied 100 times using randomly selected cells from each population, and it was tested whether the cells in the test data were correctly assigned to the relevant populations. For the RF algorithm, the number of trees was set to 500, the number of random features that needed to be used when splitting nodes in each tree was entered into the algorithm as the square root of the number of genomic features, and other parameters were left as default. In the gradient boosting algorithm, the number of trees was set to 2500, the learning rate was set to 0.001, the tree depth was set to 5, and the minimum observation count in the terminal nodes of the trees was set to 5, while other parameters were left as default. In the extreme gradient boosting, the maximum depth was set to 15, the learning rate was set to 0.01, the ratio of features in the data in each tree was set to 0.5, eta was set to 0.001, and the maximum boosting number was set to 2500 in the model. Each model was run 100 times and the outputs of each repetition of each model were recorded. At the end of each model, the effective genes in classification were listed and the most important genes seen in at least 50 repetitions were selected. The complete list of the top 100 genes from each machine learning model and the list of common genes that are defined as important genes are given in Supplementary Files (Supplementary Files 1–4).

### 2.3. Defining promoter and enhancer regions

To identify promoter and enhancer regions associated with the genes identified through ML algorithms, we used the GeneAlaCart database (https://genealacart.genecards.org/). Along with the genomic coordinates of these genes, we obtained the gene-enhancer association parameter from the same database. This parameter refers to the functional interaction between a gene and its associated enhancer or promoter regions and indicates the strength of interaction between a gene and its associated promoter and/or enhancer regions (Fishilevich et al., 2017).

### 2.4. scATAC-seq

scATAC-seq data for GBM patients were downloaded from the Gene Expression Omnibus (GEO) database with accession number GSE139136. The folder is composed of the count file containing the raw read counts, the barcode files containing cell IDs, and the peak files containing the genomic regions of the corresponding cell for each patient. There are 4 patients all of which are primary GBM and Isocitrate Dehydrogenase Wild Type (IDH-WT): G4218 (male, 64 years), G4250 (male, 73 years), G4275 (female, 52 years), and G4349 (male, 62 years). For more details, readers are referred to Guilhamon et al. (2021).

After downloading the scATAC-seq peaks and their read counts for each patient, our next step was to identify the overlapping regions between the single-cell peaks and the peaks of genes obtained through machine learning algorithms, without considering the strand orientation. Then we performed the following steps to calculate the read counts that were mapped to these overlapping regions:

1. Compute the midpoint of the promoter and/or enhancer regions identified for the genes obtained through ML algorithms.
2. Test whether the midpoint calculated in the first step falls into the corresponding peaks of GBM scATAC-seq peak data. The “*mergeByOverlaps()*” function in the IRanges package was used to identify the overlapping regions, and the read counts associated with these regions were retrieved from the scATAC-seq read counts.
3. The read counts of each gene in each cell were calculated by combining the corresponding genomic regions for each gene (i.e. taking the total sum of counts). In this step, we calculated Z-scores of each cell and cells that fall within the region below the statistical threshold of μ − 1_*_ σ are assigned a value of zero. This will allow us to have stemness scores of lowly expressed genes as zero. Additionally, since gene association power is different for each peak, we took the average of the gene association power of peaks for each gene.

At the end of these steps, a new matrix was created composed of the read counts of each gene in each cell, the importance degree of each gene obtained by machine learning methods, and the gene association power. The weights of gene importance and gene interaction strength were normalized using the “*scale()*” function and the read counts were transformed using log_2_(*x* +1).

### 2.5. Modeling the stemness score

The cancer stemness score for each cell was calculated using the following formula:


$${ATAC}_{ss}=\sum_{i\in G}w_{i}x_{i}$$

Here *ATAC_ss_* represents chromatin stemness score, *G* refers to the gene list, *w_i_* and *x_i_* are weight and total read counts of the *i^th^* gene, respectively. Weight for the *i^th^* gene, *w_i_*, is calculated by multiplying the weight derived from gene importance with the weight derived from gene association power. To scale the scores between 0 and 1, each stemness score is normalized using the following formula:


$${ATAC}_{ss}^{*}=\frac{{ATAC}_{ss}-\text{min}({ATAC}_{ss})}{\text{max}({ATAC}_{ss})-\text{min}({ATAC}_{ss})}$$

### 2.6. Labeling cells based on Neftel genes

Recent single-cell RNA-seq experiments have uncovered intratumoral heterogeneity within primary GBM, revealing a continuum of four cellular states: neural-progenitor-like (NPC: NPC1, NPC2), oligodendrocyte-progenitor-like (OPC), astrocyte-like (AC), and mesenchymal-like (MES: MES1, MES2) (Neftel et al., 2019).

We assigned cells to each of the six scRNAseq-derived cellular states (Neftel et al., 2019) using the biomarker genes that are identified in Neftel et al. (2019) for each state. For this purpose, the promoter regions of signature genes associated with each state were downloaded from the GeneAlaCart database. Then we calculated the midpoint of the promoter/enhancer regions of the signature genes identified in Neftel et al. (2019) and checked if the midpoint falls into the corresponding peaks of GBM scATAC-seq peak data. The “*mergeByOverlaps()*” function, part of the IRanges package, was utilized to identify overlapping regions, and the read counts associated with these regions were obtained from the scATAC-seq read counts. The read counts of each gene in each cell were calculated by combining the corresponding genomic regions for each gene (i.e. taking the total sum of counts), and the read counts were transformed using log_2_(*x* +1). To determine the cell type population for each state, heatmaps, which apply hierarchical clustering algorithms, were generated. These heatmaps were produced for each state separately.

### 2.7. Bulk ATAC-seq

It is stated in Guilhamon et al. (2021) that the functional differences between the stem-like cells found in different states of GBM are not fully understood, and it is unclear whether they represent distinct populations of glioma stem cells (GSCs) with unique properties that could guide therapy. To handle this, they used a combination of single-cell technologies and functional assays to identify unique dependencies associated with three glioblastoma stem cell states (reactive, constructive, and invasive) that are reflective of patient outcome. They provided their findings and the datasets where the GSC state labels for each sample and the coordinates of discriminating ATAC regions for each GSC state are given. In the present study, we incorporated the genomic regions associated with these states (as identified by bulk ATAC-seq) as stemness is significantly associated with them. Using this information, we assigned each cell from the scATAC-seq data to one of these three states.

## 3. Results and discussion

Stemness score is a measure of the “stemness” or self-renewal potential of cells, particularly in the context of cancer biology. Stem cells have the ability to divide and differentiate into various cell types and cancer stem cells are thought to be responsible for the initiation, growth, and spread of tumors. The stemness score is typically calculated using gene expression data and is based on the expression levels of genes that are associated with stem cell self-renewal and differentiation. This score can be used to classify tumors into different subtypes based on their stemness properties and may be used as a prognostic indicator or to guide treatment decisions.

**Figure 1:**
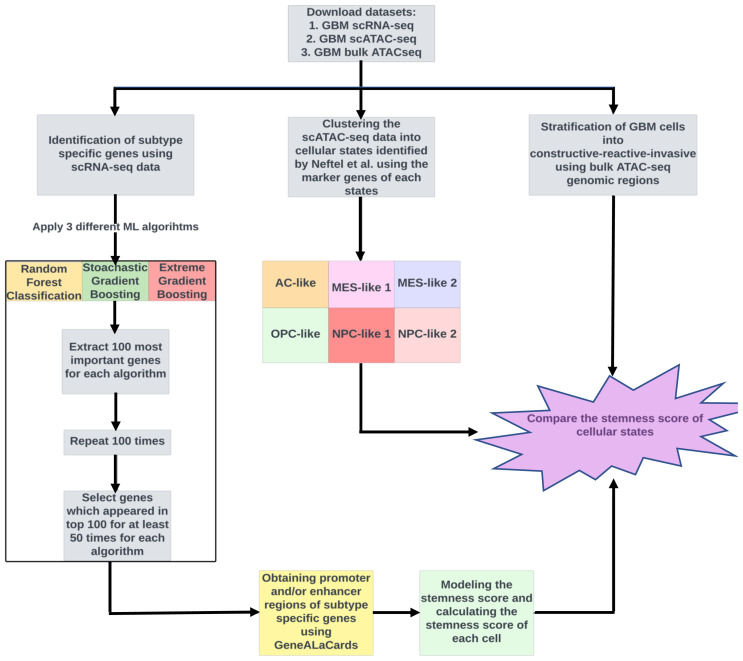
The workflow of the proposed approach. First, the datasets were downloaded for downstream analyses. Then three different machine learning algorithms were applied to identify genes that can differentiate GBM subtypes using GBM scRNAseq data. Additionally, cells were assigned into one of the cellular states identified by Neftel et al. (2019), as well as different subtypes (constructive, reactive, and invasive) using GBM scATAC-seq and bulk ATAC-seq datasets, respectively. Finally, the stemness score of each cellular state was compared.

The stemness score is a newly emerging concept in cancer research and is still being studied and refined, so its precise definition and application may vary depending on the context. Typically, transcriptomic or epigenomic data are used to quantitatively measure stemness. However, scATAC-seq data, which provide valuable information about the lineage and behavior of tumor cells, have not yet been utilized for this purpose. To address this gap, a new methodology was developed in the present study to estimate the stemness score for GBM cells using scATAC-seq data. We followed the workflow outlined in Figure 1 to calculate the stemness score for each cell and then compare the stemness of different cellular states.

**Figure 2:**
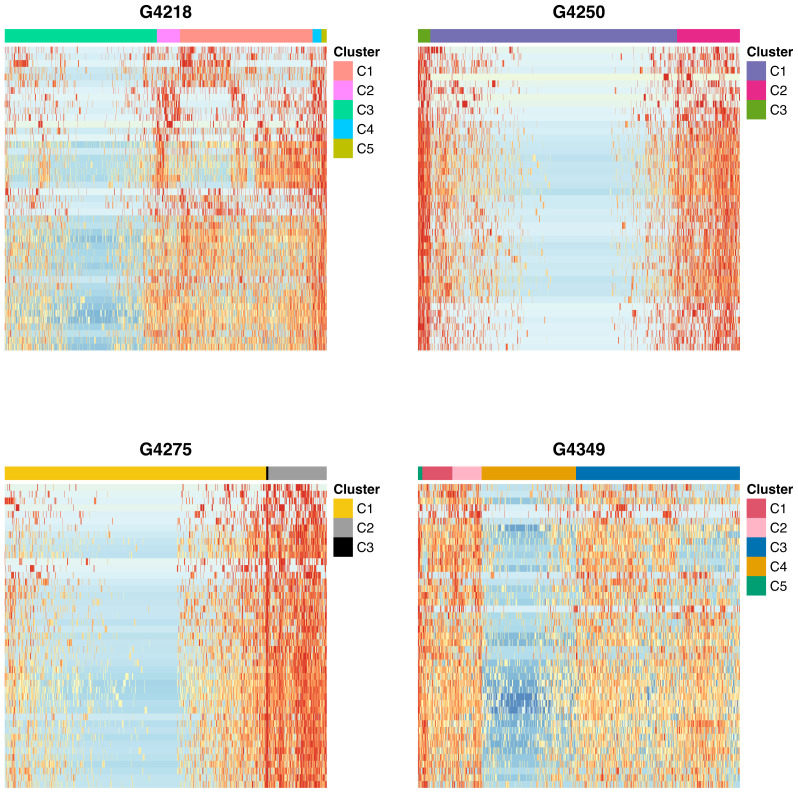
Heatmaps using MES-like 2 specific markers. The heatmaps show the expression levels of MES-like 2 specific marker genes for four patients (G4218, G4250, G4275, and G4349). The read counts were transformed using log_2_(*x* +1). Each heatmap, where rows are MES-like 2 specific marker genes and columns are cells of corresponding patient, depicts a heatmap for each patient. Clusters of cells were formed using a hierarchical clustering approach, with the number of clusters varying among patients. For instance, in the case of the second patient, three clusters were generated.

Cellular state is a term that describes the unique epigenomic character of each cell, which better explains intratumoral heterogeneity, using epigenomic and/ or transcriptomic data, and marker genes for different cellular lineages. Single-cell RNA sequencing is a commonly used method to determine and understand intratumoral heterogeneity. Using scRNA-seq, Neftel et al. (2019) reported the presence of four distinct cellular states in GBM: neural-progenitor-like (NPC-like), oligodendrocyte-progenitor-like (OPC-like), astrocyte-like (AC-like), and mesenchymal-like (MES-like) states. In their study, the MES-like and NPC-like states were further divided into two subtypes each, namely MES-like 1, MES-like 2, NPC-like 1, and NPC-like 2. Furthermore, Guilhamon et al. (2019) used scATAC-seq, which reflects chromatin accessibility, as an alternative method to determine cellular states in GBM. In the present study, we postulated that the scATAC-seq data could also be used to determine the stemness of each cell. More specifically, we used signature genes of each cellular state to identify the promoter regions of these genes and calculated chromatin accessibility. We then performed hierarchical clustering to observe the clustering structure and therefore determine which cells belong to the corresponding cellular state. For instance, Figure 2 displays heatmaps for each patient when MES-like 2 specific marker genes were used. According to Figure 2, we categorized cells into 5 different clusters for the first and fourth patients (i.e. G4218 and G4349), while we categorized cells into 3 different clusters for the second and third patients (i.e. G4250 and G4275). The cells with high chromatin accessibility at MES-like 2 marker genes were identified as MES-like 2 cells. Additionally, we refer readers to the supplementary data for heatmaps of other cellular states for each patient (Supplementary Figures S1–S5).

**Figure 3:**
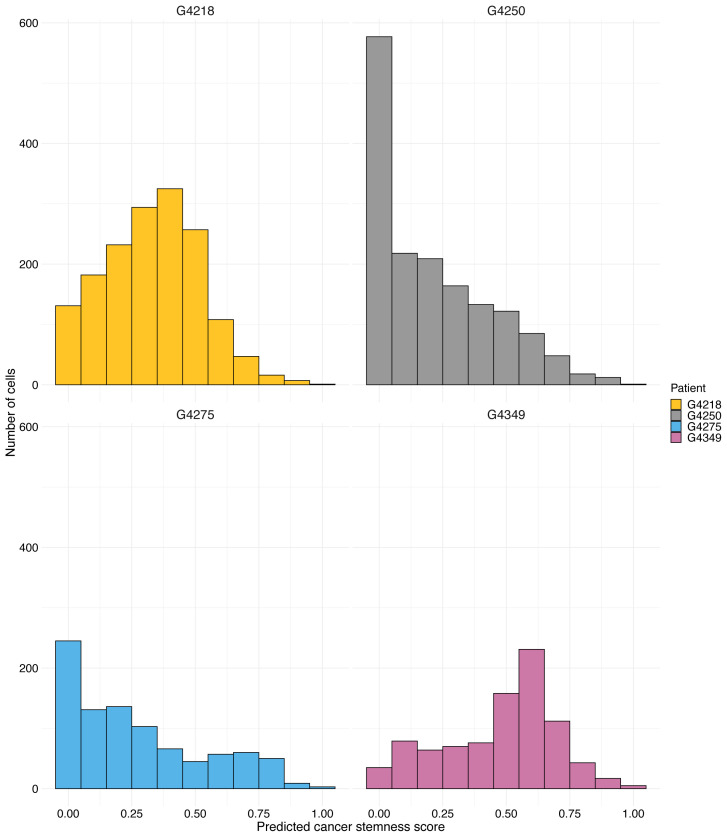
Stemness score of each cell for each patient. Bar plots show the stemness score of each cell for four patients (G4218, G4250, G4275, and G4349). The x-axis of each bar chart represents the predicted stemness score, while the y-axis indicates the frequency of cells with that score.

To estimate the stemness score for each cell, we first identified subtype-specific genes using scRNA-seq data available at https://www.ncbi.nlm.nih.gov/bioproject/ with the accession number PRJNA324289. We applied three ML algorithms to identify genes that were predictive of the four different cellular states of GBM cells, namely qNSC, aNSC^low^, aNSC^high^, and NPC. After identifying state-specific genes, we extracted promoter and/or enhancer regions of those genes using GeneALaCards. We used this information, combined with gene importance as determined by ML algorithms, to model and estimate the stemness score of each cell. Figure 3 displays the stemness score distribution of cells for each patient, revealing that many cells for each patient had a stemness score of either zero or below 0.5, as expected. To demonstrate the proportion of the cells in each group with <0.5 stemness score (low stemness) and >0.5 (high stemness), pie charts are also provided (Supplementary Figure S6). Our findings indicate that our novel algorithm based on scATAC-seq data can predict stemness scores in accordance with the literature where a minor fraction of cells in a GBM tumor have high stemness scores (Suva and Tirosh, 2020).

Primary GBM is composed of distinct cellular states, with stem-like cells present across these states. Wang et al. (2019) showed that GBM cells can be positioned along a single axis of variation, ranging from proneural to mesenchymal transcriptional profiles. More specifically, stemness-associated genes were at the extremes of this axis, indicating the presence of stem-like cells in different states of GBM. The functional properties and tumor-initiating capabilities of these stem-like cells, as well as their unique features, remain to be fully understood. It is crucial to establish whether these stem-like cells in different GBM states represent functionally distinct populations with unique properties. This understanding would guide therapeutic advancements for GBM treatment. To address this issue, Guilhamon et al. (2021) combined single-cell technologies to define the composition of glioma stem cells in primary GBM. They also performed functional assays to identify the unique dependencies of GSCs, which reflect invasive, constructive, and reactive states. These distinct states of GSCs are associated with patient outcomes, providing valuable insights for developing targeted therapies and improving patient prognosis. The subset classified as invasive GSCs indicates the worst prognosis, whereas the reactive subset shows a more favorable prognosis.

**Figure 4:**
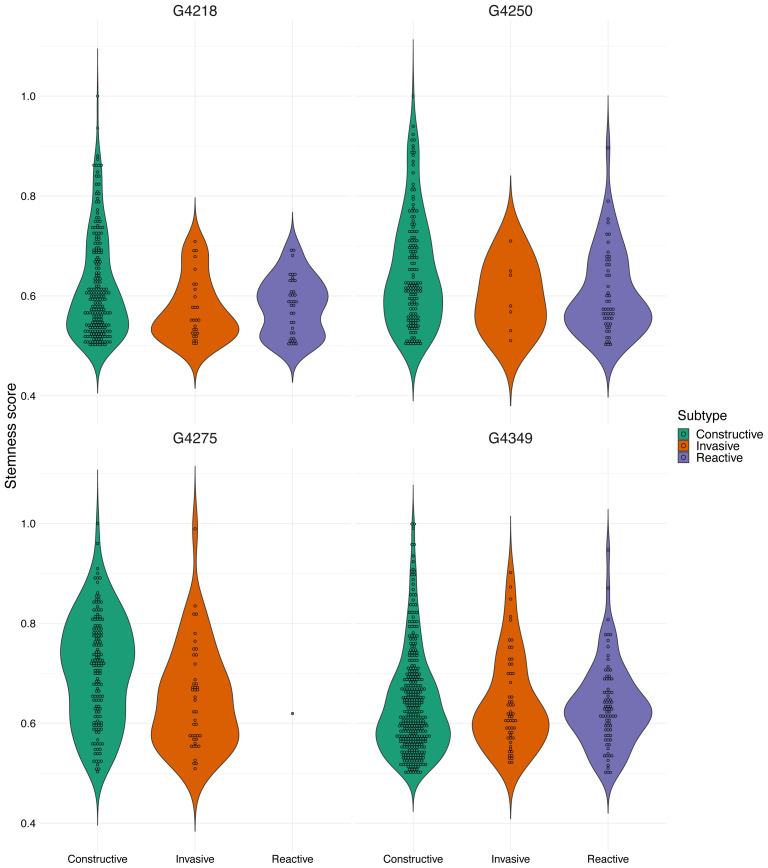
Violin plots for each of the four patients (G4218, G4250, G4275, and G4349). Each plot represents the stemness score for each of the three subtypes (i.e. constructive, invasive, and reactive).

**Figure 5:**
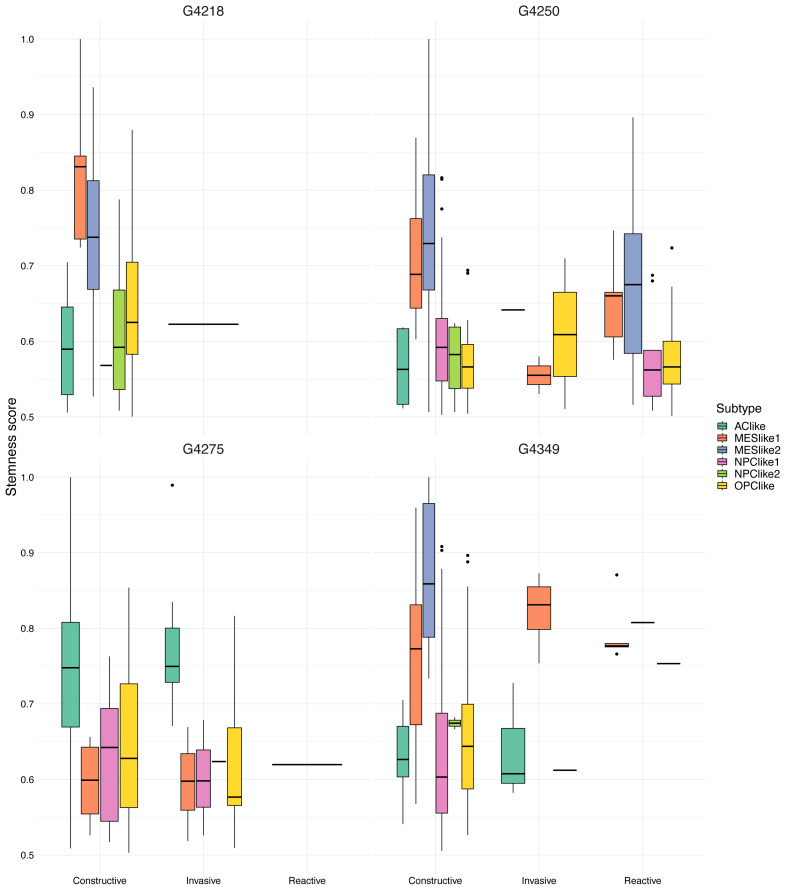
Predicted stemness score for each subtype. Bar plots depict the predicted stemness scores for GBM subtypes identified by Neftel et al. (2019) and classified into constructive, invasive, and reactive groups for each patient.

In the present study, we also utilized the classification suggested by Guilhamon et al. (2021), namely constructive, reactive, and invasive, to compare the distribution of stemness scores across these proposed functional states. Therefore, when labeling cells with one of these three states, we filtered out those with stemness scores below 0.5. After filtering, for each patient we created violin plots, which display the distribution of the stemness scores for each state (Figure 4). We then combined the constructive, reactive, and invasive groups with the subtypes suggested by Neftel et al. (2019) and compared the stemness score of each cellular state. Figure 5 depicts the predicted stemness scores for each cellular state based on their respective groups. These results show that the stemness scores of MES-like cells are higher than those of other cellular states, particularly for constructive cells, in 3 out of 4 patients. Our findings are consistent with those of previous studies, in the sense that the mesenchymal subtype (MES-like) has the highest stemness score, in accordance with stronger association of GBM mesenchymal cells with tumor initiation capacity, resistance to chemo-/radiotherapy, worse prognosis, etc. (Behnan et al., 2019).

Due to the lack of ground truth in our dataset, our analysis is limited in terms of establishing benchmarks or reference points or conducting validation on independent datasets. Our findings, however, demonstrate that our innovative algorithm, utilizing scATAC-seq data, effectively predicts stemness scores, measurements of the stemness of each glioma cell. In contrast to other methods that predict stemness scores based on transcriptomic or epigenomic data, our approach utilizes scATAC-seq data to characterize the stemness features of glioblastoma cells.

## 4. Conclusion

Overall, our study highlights the usefulness of scATAC-seq in assessing GBM heterogeneity and identifying cells characterized by their high stemness properties. This novel approach provides valuable insights into the stemness landscape of GBM, offering potential avenues for improved therapeutic strategies targeting these high-risk cell populations.

## Supplementary Data

Supplementary Figure S1 Heatmaps using NPC-like 1 specific markers. The heatmaps show the expression levels of NPC-like 1 specific marker genes for four patients (G4218, G4250, G4275, and G4349). Each heatmap, where rows are NPC-like 1 specific marker genes and columns are cells of the corresponding patient, depicts a heatmap for each patient.

Supplementary Figure S2 Heatmaps using NPC-like 2 specific markers. The heatmaps show the expression levels of NPC-like 2 specific marker genes for four patients (G4218, G4250, G4275, and G4349). Each heatmap, where rows are NPC-like 2 specific marker genes and columns are cells of the corresponding patient, depicts a heatmap for each patient.

Supplementary Figure S3 Heatmaps using OPC-like specific markers. The heatmaps show the expression levels of OPC-like specific marker genes for four patients (G4218, G4250, G4275, and G4349). Each heatmap, where rows are OPC-like specific marker genes and columns are cells of the corresponding patient, depicts a heatmap for each patient.

Supplementary Figure S4 Heatmaps using AC-like specific markers. The heatmaps show the expression levels of AC-like specific marker genes for four patients (G4218, G4250, G4275, and G4349). Each heatmap, where rows are AC-like specific marker genes and columns are cells of the corresponding patient, depicts a heatmap for each patient.

Supplementary Figure S5 Heatmaps using MES-like 1 specific markers. The heatmaps show the expression levels of MES-like 1 specific marker genes for four patients (G4218, G4250, G4275, and G4349). Each heatmap, where rows are MES-like 1 specific marker genes and columns are cells of the corresponding patient, depicts a heatmap for each patient.

Supplementary Figure S6 Pie charts of proportion of cells in each group for four patients (G4218, G4250, G4275, and G4349). The pie charts illustrate the distribution of cells based on their stemness levels, categorized as low stemness and high stemness. The percentages corresponding to each category are provided within the figures. Cells with a stemness score of less than 0.5 are classified as low stem cells, while those with a score greater than 0.5 are classified as high stem cells.
