# Supplementary figures and images for: StemnesScoRe: an R package to estimate the stemness of glioma cancer cells at single-cell resolution
Source: Turk J Biol. 2023 Dec 15;47(6):383–92. doi: 10.55730/1300-0152.2672 (PMC11045207; doi:10.55730/1300-0152.2672)

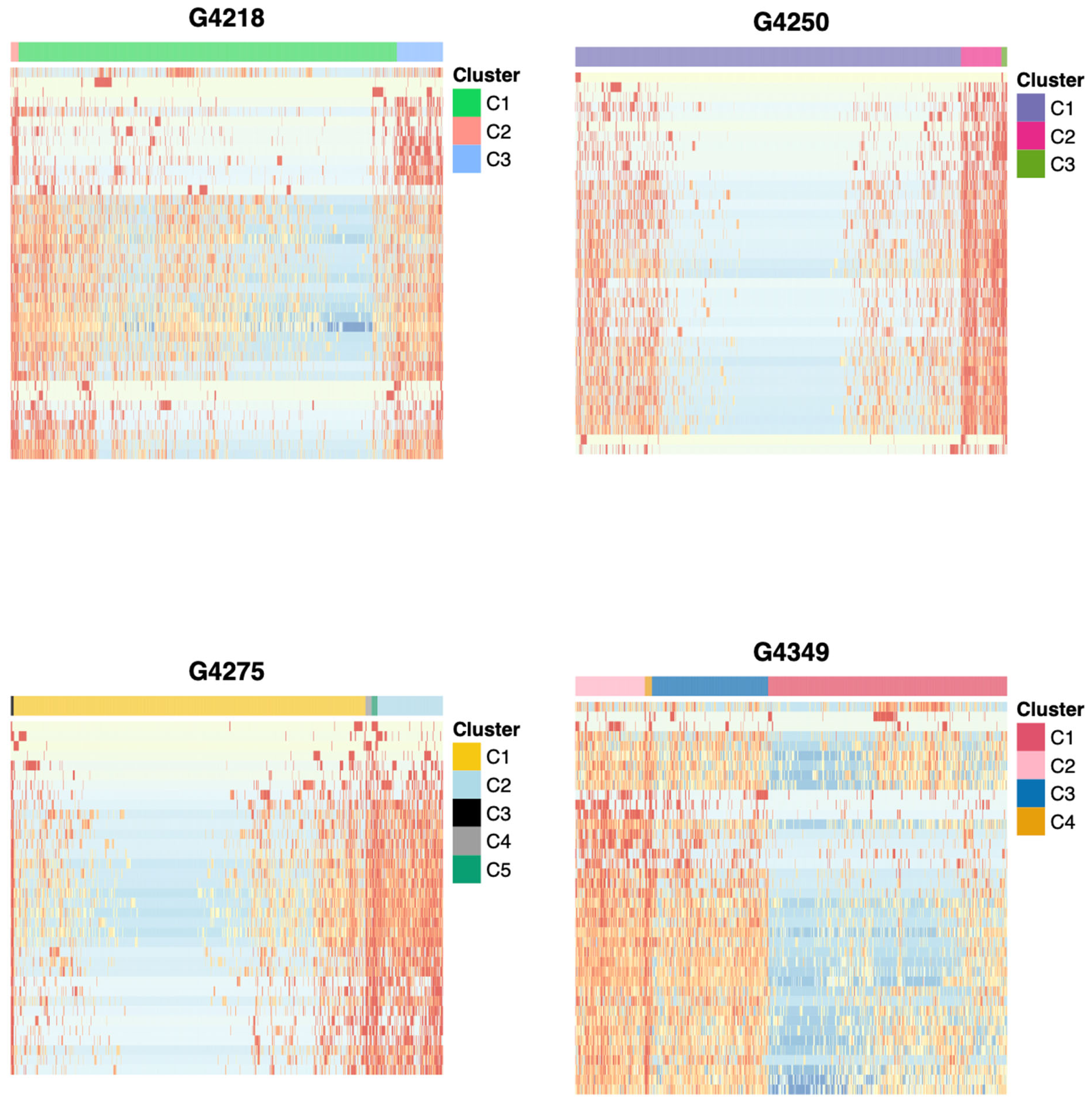

Supplement: Supplementary Figure S1 — Heatmaps using NPC-like 1 specific markers. The heatmaps show the expression levels of NPC-like 1 specific marker genes for four patients (G4218, G4250, G4275, and G4349). Each heatmap, where rows are NPC-like 1 specific marker genes and columns are cells of the corresponding patient, depicts a heatmap for each patient. [file tjb-47-06-383s1.tif]

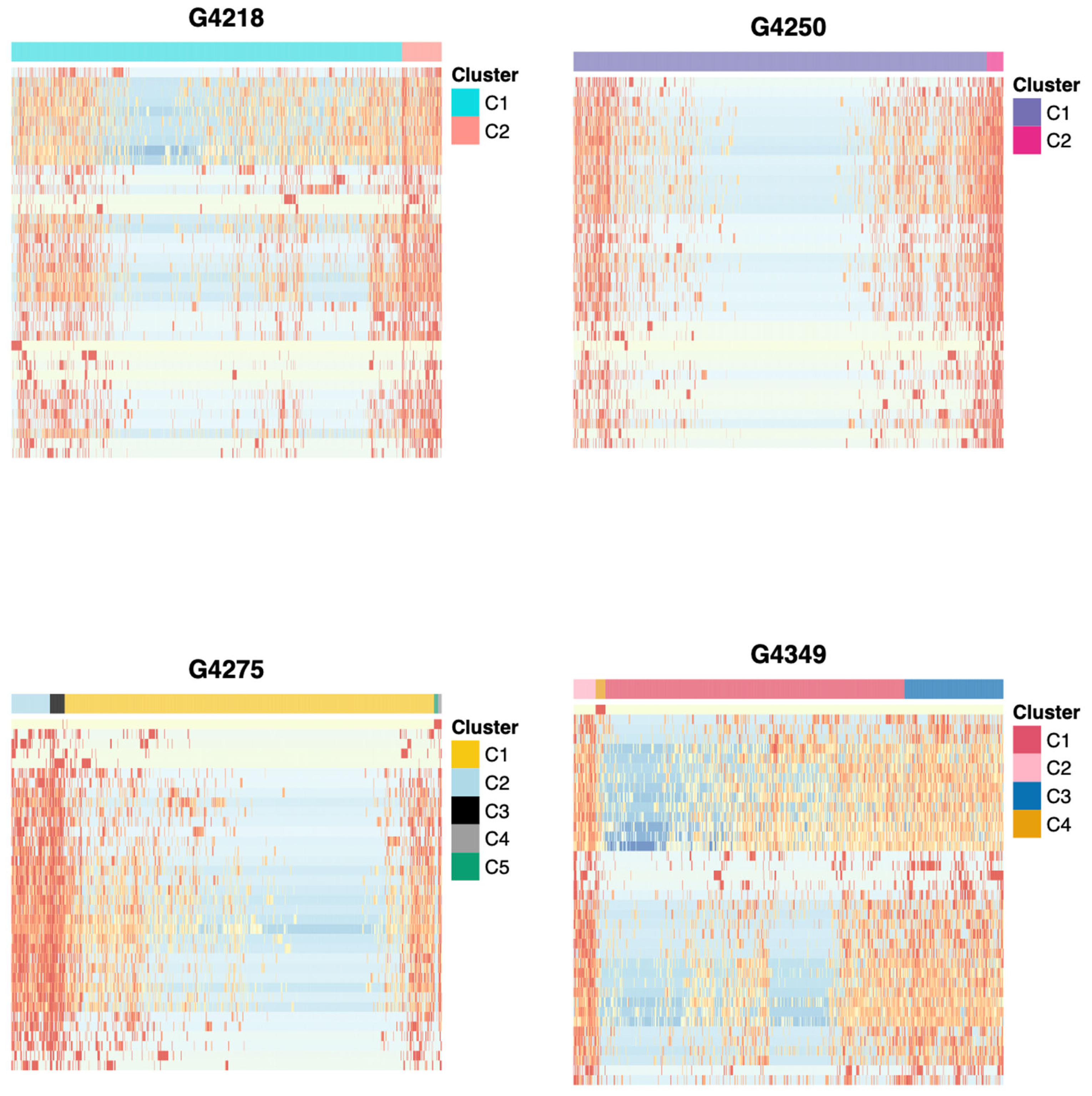

Supplement: Supplementary Figure S2 — Heatmaps using NPC-like 2 specific markers. The heatmaps show the expression levels of NPC-like 2 specific marker genes for four patients (G4218, G4250, G4275, and G4349). Each heatmap, where rows are NPC-like 2 specific marker genes and columns are cells of the corresponding patient, depicts a heatmap for each patient. [file tjb-47-06-383s2.tif]

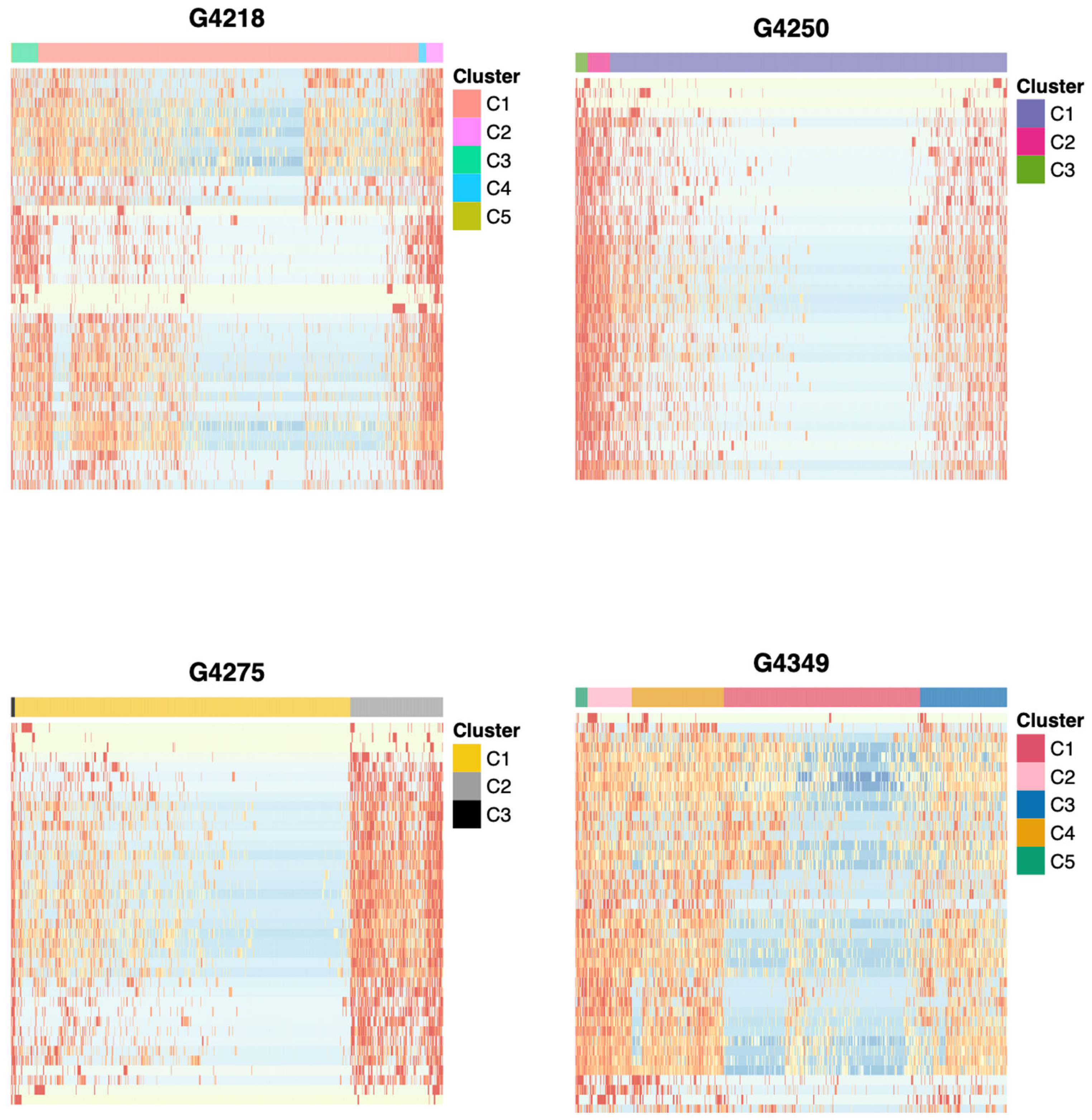

Supplement: Supplementary Figure S3 — Heatmaps using OPC-like specific markers. The heatmaps show the expression levels of OPC-like specific marker genes for four patients (G4218, G4250, G4275, and G4349). Each heatmap, where rows are OPC-like specific marker genes and columns are cells of the corresponding patient, depicts a heatmap for each patient. [file tjb-47-06-383s3.tif]

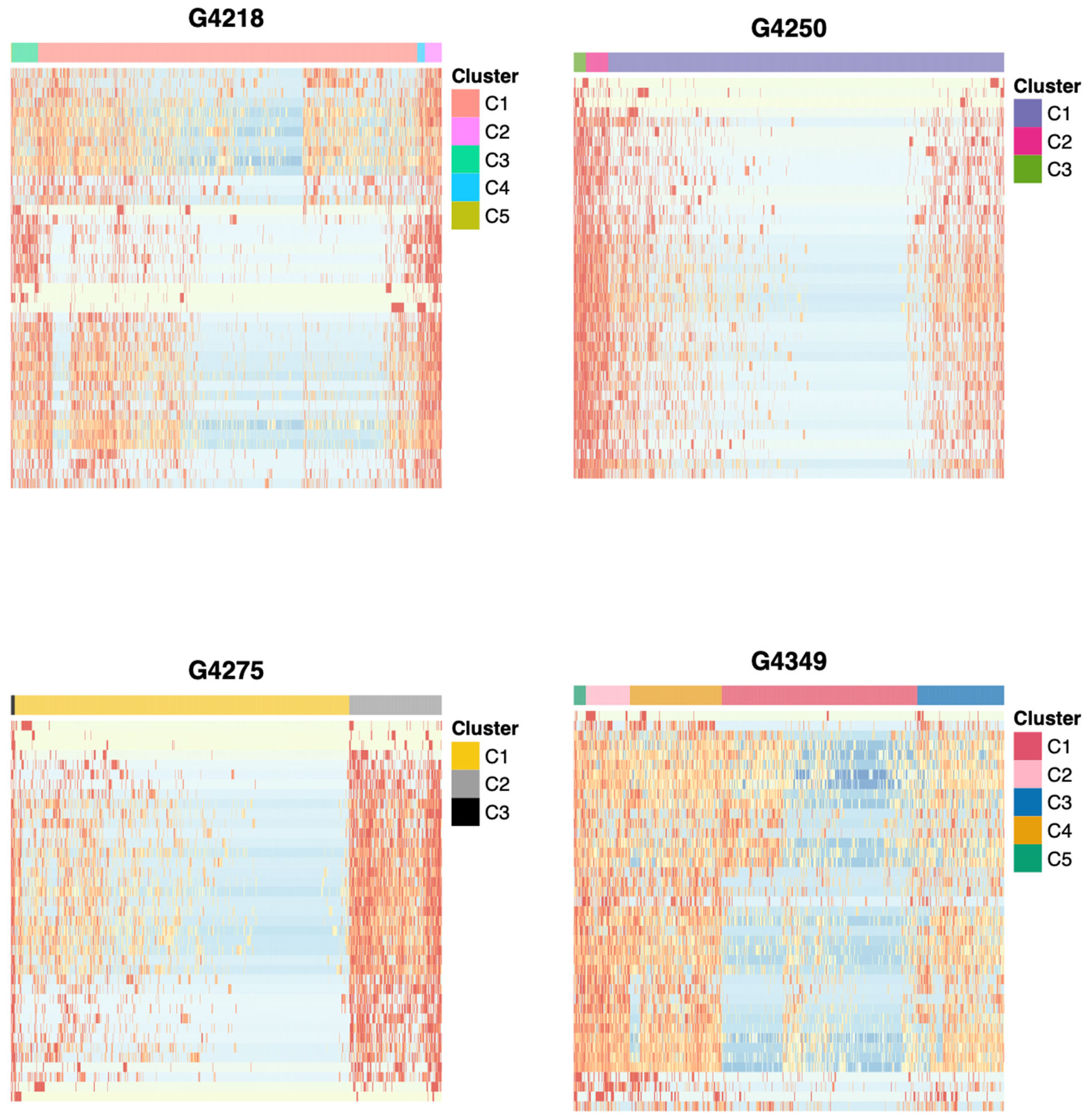

Supplement: Supplementary Figure S4 — Heatmaps using AC-like specific markers. The heatmaps show the expression levels of AC-like specific marker genes for four patients (G4218, G4250, G4275, and G4349). Each heatmap, where rows are AC-like specific marker genes and columns are cells of the corresponding patient, depicts a heatmap for each patient. [file tjb-47-06-383s4.tif]

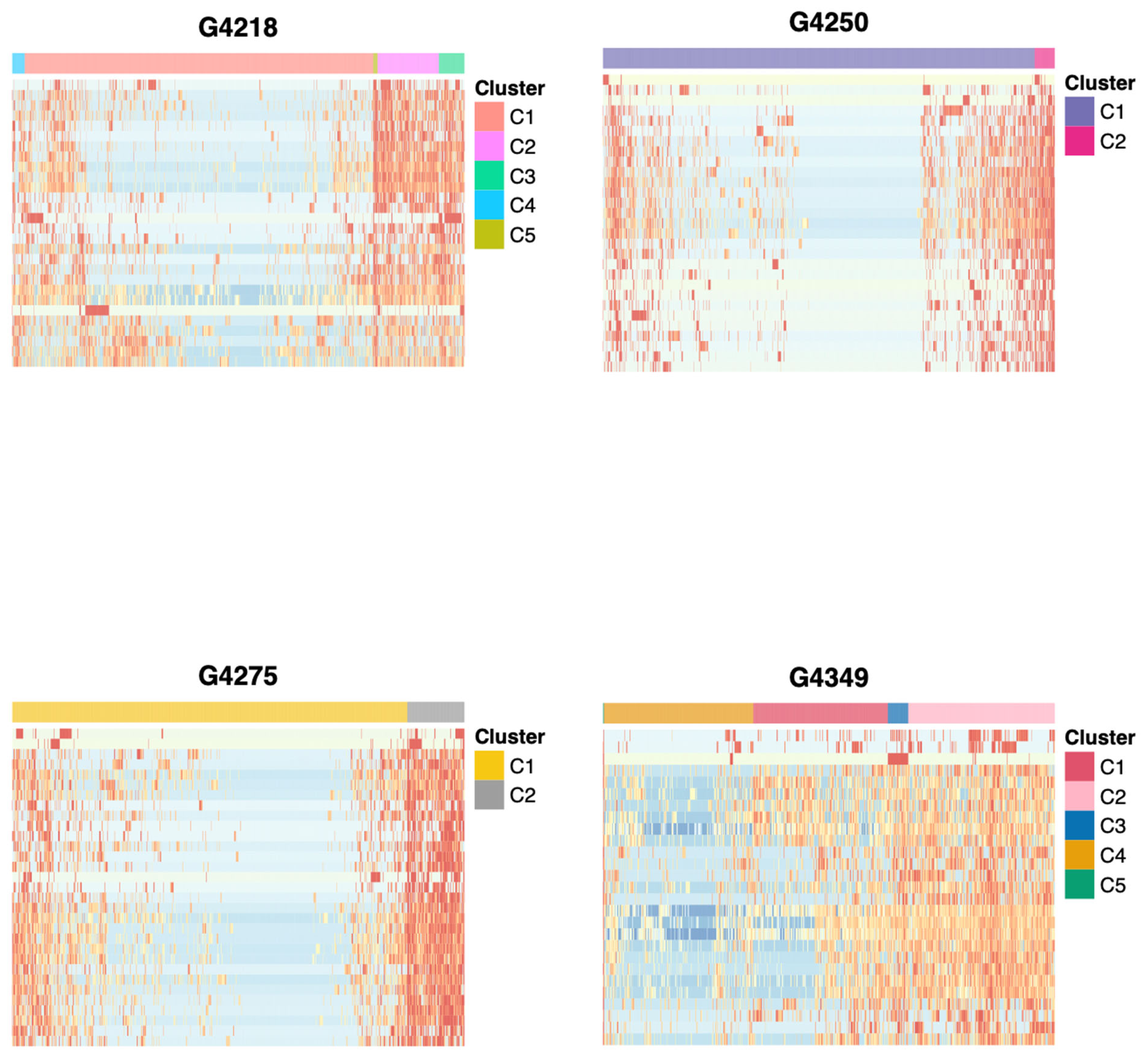

Supplement: Supplementary Figure S5 — Heatmaps using MES-like 1 specific markers. The heatmaps show the expression levels of MES-like 1 specific marker genes for four patients (G4218, G4250, G4275, and G4349). Each heatmap, where rows are MES-like 1 specific marker genes and columns are cells of the corresponding patient, depicts a heatmap for each patient. [file tjb-47-06-383s5.tif]

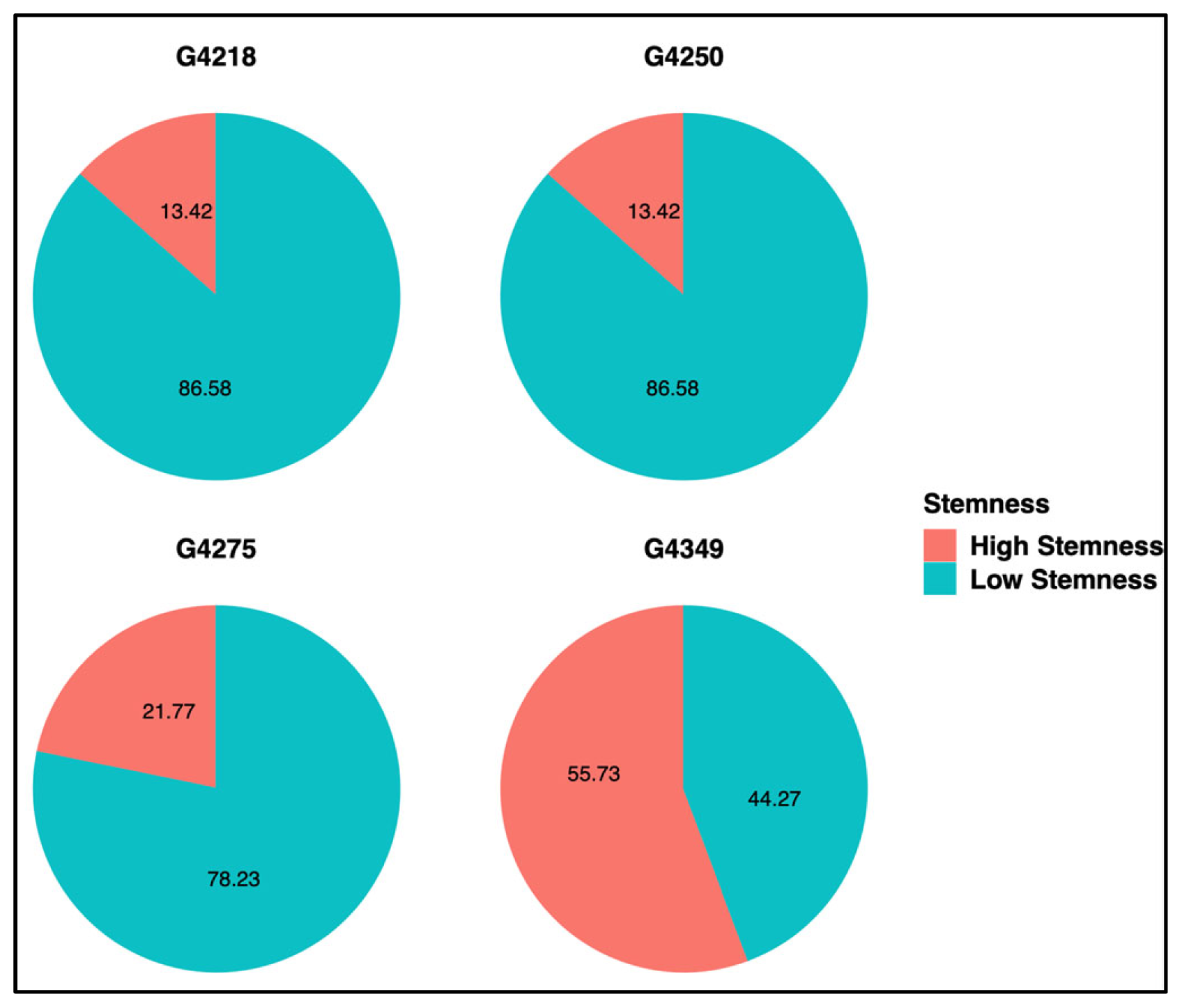

Supplement: Supplementary Figure S6 — Pie charts of proportion of cells in each group for four patients (G4218, G4250, G4275, and G4349). The pie charts illustrate the distribution of cells based on their stemness levels, categorized as low stemness and high stemness. The percentages corresponding to each category are provided within the figures. Cells with a stemness score of less than 0.5 are classified as low stem cells, while those with a score greater than 0.5 are classified as high stem cells. [file tjb-47-06-383s6.tif]
